# An Updated Meta-Analysis of Randomized Controlled Trials Comparing Direct Oral Anticoagulants Against Warfarin for Left Ventricular Thrombus Resolution

**DOI:** 10.3390/jcm14196735

**Published:** 2025-09-24

**Authors:** Joseph Magdy, Maggie He, Sacchin Arockiam, Nanami Harada, Stephen B. Wheatcroft, Heerajnarain Bulluck

**Affiliations:** 1 Yorkshire Heart Centre, Leeds General Infirmary, Leeds Teaching Hospitals NHS Trust, Leeds LS1 3EX, UK; 2 Leeds Institute of Cardiovascular and Metabolic Medicine, University of Leeds, Leeds LS1 3EX, UK

**Keywords:** left ventricular thrombus, direct oral anticoagulants, vitamin K antagonists, embolic complications, bleeding

## Abstract

**Background**: Left ventricular thrombus (LVT) remains a well-recognized complication following myocardial infarction (MI). Whilst vitamin K antagonists (VKAs) have traditionally been the cornerstone of management, direct oral anticoagulants (DOACs) have been increasingly utilized despite limited data to support this. We sought to perform an up-to-date meta-analysis of all randomized controlled trials (RCTs) comparing DOACs to VKAs for LVT resolution. **Methods**: A systematic search of major scientific databases was performed to identify RCTs published until May 2025. The primary efficacy endpoint was complete LVT resolution at 3 months. The risk ratio (RR) and 95% confidence intervals (CIs) of the individual RCTs were pooled via the inverse-variance method and random-effects model. **Results**: Seven RCTs involving 554 patients with a mean age of 54 years were included in the meta-analysis. At 3 months, there was no difference in the rate of LVT resolution between those in the DOAC arm and the warfarin arm (86% vs. 81%, RR 1.01 [95%CI 0.93–1.10], *p* = 0.76). There was low heterogeneity at I^2^ = 15%. There was no difference in major or clinically significant bleeding or in the composite of stroke or thromboembolic complications, although the 95%CIs were wide. **Conclusions**: DOACs appear to be comparable to warfarin in achieving LVT resolution at 3 months. These findings support the consideration of DOACs as alternatives to VKAs in selected patients for LVT resolution. Further adequately powered trials and head-to-head comparisons between DOACs are required to confirm their safety.

## 1. Introduction

Left ventricular thrombus (LVT) is a well-recognized complication of myocardial infarction (MI) [1]. Patients with reduced left ventricular ejection fraction (LVEF), anterior myocardial infarction, elevated troponin levels and inflammatory markers have been identified as risk factors for developing LVT [2]. Despite advancements in reperfusion techniques, LVT is still observed post-MI in nearly 20% of patients with anterior ST-segment elevation myocardial infarction (STEMI) and impaired LV function by cardiac magnetic resonance (CMR) [3].

In addition to acute MI, dilated cardiomyopathy (DCM) is another important substrate for LVT formation. Historically, echocardiographic series reported prevalence rates between 10 and 30% [4,5]. However, more recent cohort studies suggest a lower prevalence rate of 2.3% among over 3000 patients with DCM at a single tertiary center in Beijing [6] and 7.1% in a multicenter analysis of 1267 patients with non-ischemic cardiomyopathy [7]. Randomized data specific to DCM-associated LVT are still lacking, as most RCTs involving anticoagulation for LVT focus on post-MI populations, with few studies reporting outcomes specifically for patients with DCM. The burden of LVT formation is associated with increased risk of stroke, distal embolization and other major cardiovascular events [8].

Traditionally, vitamin K antagonists (VKAs), such as warfarin, have been the cornerstone of LVT management, guided by evidence extrapolated from pre-reperfusion era studies and real-world experience. However, limitations including narrow therapeutic window, food and drug interactions, and variable time in therapeutic range pose challenges to optimal management. The availability of direct oral anticoagulants (DOACs) has transformed anticoagulation in other clinical settings, offering predictable pharmacokinetics, fewer interactions, and no requirement for routine monitoring. To date, in the absence of large, randomized controlled trials (RCTs), the comparative effectiveness of DOACs remains uncertain [1].

Despite this, DOACs are increasingly prescribed off-label for LVT, supported by small RCTs and meta-analyses of predominantly observational studies suggesting comparable efficacy and safety to VKAs [9,10,11]. A recent meta-analysis of RCTs only showed no difference in LVT resolution between DOAC and warfarin, although the sample size was small [10]. Recently, RIVAWAR trial result was presented at the American College of Cardiology (ACC) 2025 annual congress. This single-center, open-label, randomized study including 261 patients, showed that by 12 weeks, there were no significant differences in LVT resolution between rivaroxaban and warfarin [12].

Therefore, we aimed to perform an up-to-date meta-analysis of all RCTs comparing DOAC against warfarin for the treatment of LVT resolution.

## 2. Materials and Methods

This meta-analysis was conducted following the Preferred Reporting Items for Systematic Reviews and Meta-analyses (PRISMA) guidelines [13].

### 2.1. Eligibility Criteria

We included all RCTs enrolling patients with a confirmed diagnosis of LVT that compared direct oral anticoagulants (DOACs) versus vitamin K antagonists (VKAs) and reported LVT resolution during follow-up, whether published as full-text papers or abstracts.

### 2.2. Search Strategy

A systematic search was performed using PubMed/MEDLINE, Ovid/Embase, and the Cochrane Central Register of Controlled Trials (CENTRAL) to identify RCTs published in English up to May 2025. The search strategy combined terms including “direct oral anticoagulants,” “vitamin K antagonists,” “randomized controlled trials,” and “left ventricular thrombus”.

### 2.3. Study Selection

Two authors (MH, SA) independently screened titles and abstracts for eligibility and performed full-text review and data extraction. Discrepancies were resolved by consensus with a third reviewer (HB). As this study utilized previously published data, institutional review board approval and informed consent were not required. Eligible RCTs included in this meta-analysis are summarized in Table 1. 

### 2.4. Data Extraction and Quality Assessment

**Figure 1:**
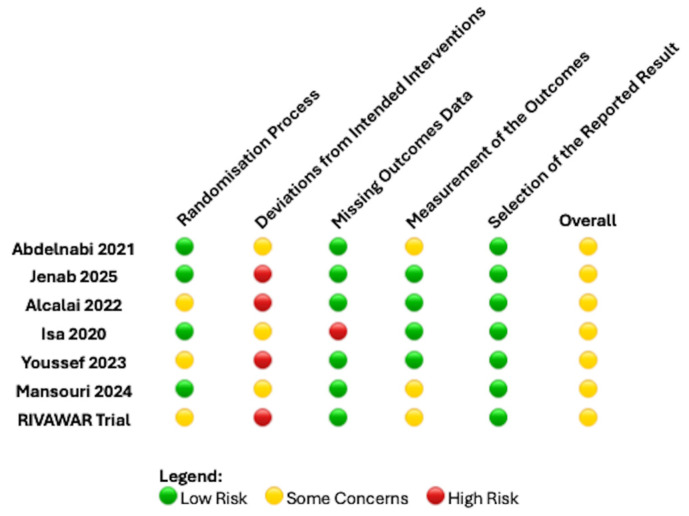
Risk of bias summary by Cochrane risk assessment tool for the RCTs included [11,12,15,16,17,18,19].

Extracted variables included study design, patient population, anticoagulant regimen (DOAC vs. VKA), inclusion and exclusion criteria, clinical outcomes, and duration of follow-up. Risk of bias for individual studies was assessed in accordance with the Cochrane risk-of-bias assessment tool (Figure 1) [14].

**Table 1:** Characteristics of studies included.

Study	Total Number of Patients and Country	RCT Type and Intervention	Inclusion and Exclusion Criteria	Follow-Up Duration	Endpoints
Isa et al. 2020 [17]	*n* = 27 Malaysia	Pilot, prospective, single-center, randomized, single-blinded outcome study Apixaban 5 mg BD or 2.5 mg BD for 12 weeks, based on recommendation Warfarin with initial heparin infusion, aiming for a target INR of 2–3	Inclusion criteria: Age 18–80 years old LVT confirmed by 2D echocardiography by two operators HAS-BLED score < 3 Exclusion criteria: Episodes of major bleeding in the past 6 months History of intracranial bleeding or large ischemic stroke Advanced renal and liver disease on cardiac devices Clinically unstable or in shock	15 weeks (Echocardiography 12 weeks)	Primary outcome: 12 weeks percentage of LVT mean size reduction or total resolution Secondary outcomes: All-cause mortality Ischemic stroke Worsening heart failure
Abdelnabi et al. 2021 [19]	*n* = 79 Egypt Bulgaria	Prospective, open-label, multi- center, RCT Rivaroxaban 20 mg daily Warfarin with initial enoxaparin until reaching target INR 2–3	Inclusion criteria >18 years old Evidence of LVT assessed by TTE Exclusion criteria CrCl < 50 mL/min	1, 3, 6 months	Primary outcomes: Presence or absence of LVT as assessed by 2D transthoracic echocardiography Secondary outcomes: Stroke or systemic embolism Major bleeding
Alcalai et al. 2022 [18]	*n* = 35 Israel	Multicentre, national, randomized open-label non-inferiority clinical trial Apixaban 5 mg BD or 2.5 mg BD for 3 months dose based on recommendations Warfarin targeting INR 2–3	Inclusion criteria: Age 18–100 years old Patients with evidence of LVT assessed by TTE Acute MI in last 3 months prior to enrolment Exclusion criteria: Contraindication for chronic anticoagulation Severe renal failure (CrCl < 15 mL/min) Patients with other indications for chronic anticoagulation (e.g., AF, pulmonary embolism) Patients with technically limited 2D-TTE	3 months	Primary outcome: Presence and dimensions of LVT as assessed by 2D echocardiography Secondary outcomes: Stroke or systemic embolism Major bleeding All-cause mortality
Youssef et al. 2023 [15]	*n* = 50 Saudi Arabia	Open-label RCT Apixaban 5 mg BD Warfarin targeting INR 2–3	Inclusion criteria: Age 18–80 years old Acute (within a week) or recent (within a month) anterior wall MI Evident LVT on TTE Naïve to oral anticoagulants Stable to start oral anticoagulants Exclusion criteria: Other indications for oral anticoagulants Patients with contraindications for oral anticoagulants Right ventricular thrombus or atrial thrombus History of confirmed stroke or other systemic embolization within the previous six months High bleeding risk Severe renal impairment Patients with expected difficulties to follow the INR strictly	1, 3, 6 months	Primary outcome: Resolution of LVT in 3 months Secondary outcomes: Resolution of LVT in 6 months Safety outcome: MACE or any relevant bleeding according to the BARC classification
Mansouri et al. 2024 [16]	*n* = 52 Iran	open- label non-inferiority RCT Rivaroxaban 20 mg daily Warfarin 5 mg loading dose aiming INR 2–3	Inclusion criteria: Age > 18 years old admitted with a diagnosis of ACS undergoing percutaneous coronary intervention (PCI) LV apical thrombus detected in echocardiography Exclusion criteria: eGFR < 30 mL/min/1.73 m Concomitant major trauma or active bleeding Uncontrolled hypertension Prior hemorrhagic stroke History of major ischemic stroke Current oral anticoagulation for other indications (e.g., AF, VTE) Acute liver disease Pregnancy Inherited or acquired bleeding disorder	3 months	Primary outcome: Resolution of LVT on TTE in 3 months Secondary outcomes: Bleeding Systemic embolic events Rehospitalization MACE Echocardiographic measures focusing on changes in thrombus size, mobility, and morphology
Jenab et al. 2025 [11]	*n* = 50 Iran	Pilot, open-label, parallel-group RCT with a 1:1 allocation ratio, concealed allocation sequences, and blinded outcome assessments Rivaroxaban (15 mg daily) plus clopidogrel (75 mg daily) plus aspirin (80 mg daily, only during the first 7 days) Warfarin overlapping with enoxaparin, until reaching an INR goal of 2.0–2.5, plus clopidogrel (75 mg daily plus aspirin (80 mg daily, only during the first 7 days)	Inclusion criteria: Age 18–80 years old Admission with acute STEMI within the previous 2 weeks Acute LVT confirmed by non-contrast 2D TTE Willing to participate Exclusion criteria: History of MPHV, APS and rheumatic heart disease Active bleeding Cardiogenic shock eGFR < 30 mL/min Liver failure Other indications for chronic anticoagulation Sensitivity or intolerance to rivaroxaban or warfarin	3 months	Primary outcome: 3-month non-contrast 2D TTE-based complete LVT resolution Other outcomes: SSE at 3 months MACE) at 3 months All-cause death at 3 months Main safety outcomes: Major bleeding events based on ISTH definition at 3 months
RIVAWAR trial [12]	*n* = 261 Pakistan	Investigator initiated, single-center trial, open-label, RCT with a 2:1 allocation ratio Rivaroxaban 20 mg once daily Warfarin targeting INR 2–3	Inclusion criteria: Age > 18 years old Patients of ACS with LVT Hemodynamically stable Willing to participate Exclusion criteria: Prior history of cardiomyopathy Anticoagulant contraindications Prior history of stroke with residual neurological deficit Valvular atrial fibrillation Pregnancy Unable to consent Deranged liver function tests (LFTS) CrCl < 50 mL/min	3 months	Primary outcome: presence of LVT on TTE at 12 weeks post-randomization Secondary outcome: All-cause mortality Ischemic stroke Major bleeding

CrCl: creatinine clearance; LVT: left ventricular thrombus; TTE: transthoracic echocardiography; MACE: major adverse cardiovascular events; BARC: bleeding Academic Research Consortium; MPHV: mechanical prosthetic heart valve; RCT: randomized controlled trial; SSE: stroke and systemic emboli.

### 2.5. Endpoints

The primary efficacy endpoint was complete resolution of LVT at 3 months. Secondary endpoint of interest was the incidence of major or clinically significant bleeding events as reported by the individual RCTs and the occurrence of stroke or thromboembolic complications.

### 2.6. Statistical Analysis

Statistical analyses were conducted using RevMan version 5.4 (Cochrane Collaboration, Nordic Cochrane Centre). Pooled risk ratios (RRs) with 95% confidence intervals (CIs) were calculated using the inverse-variance method and a random-effects model to account for variability across trials. Heterogeneity across trials was assessed using the I^2^ statistic, with thresholds of 25%, 50%, and 75% denoting low, moderate, and high heterogeneity, respectively. To estimate the comparative efficacy and safety of apixaban versus rivaroxaban in the absence of direct head-to-head RCTs, we performed an adjusted indirect comparison using the Bucher method. Specifically, we compared apixaban and rivaroxaban indirectly through their respective comparisons with the shared control group (warfarin). A two-sided *p*-value < 0.05 was considered statistically significant.

## 3. Results

**Figure 2:**
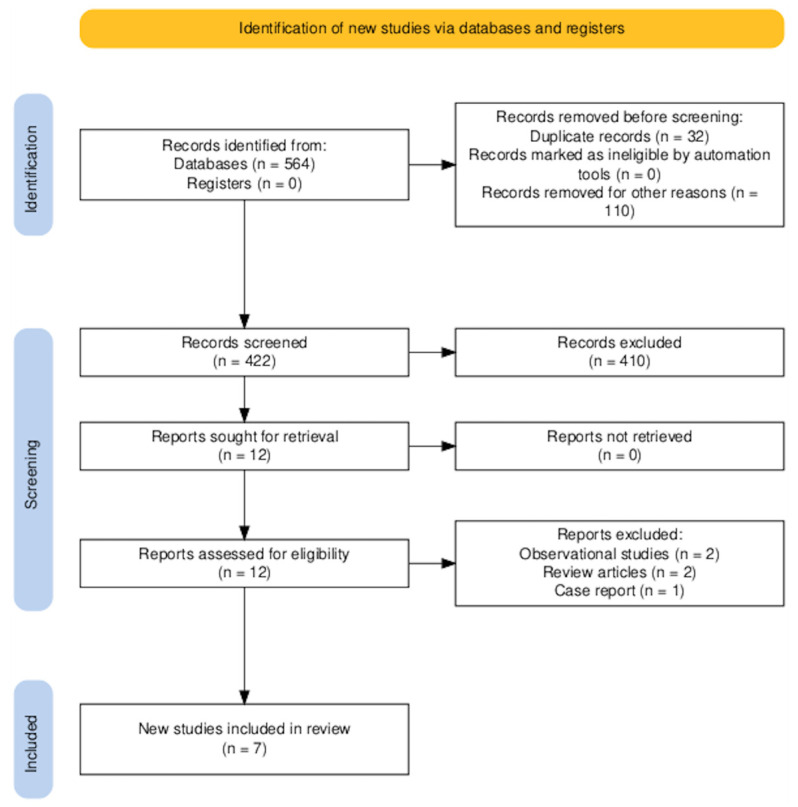
PRISMA flow diagram of the study selection process.

The PRISMA diagram is shown in Figure 2. A total of 564 records were identified through database searches. No additional records were retrieved from trial registries or other sources. Prior to the screening phase, 32 duplicate entries were removed. An additional 110 records were excluded for reasons unrelated to duplication—such as non-English language, conference abstracts without full texts, or studies unrelated to the research question—leaving 422 records for title and abstract screening.

Of these, 410 were excluded based on title and abstract review, predominantly due to irrelevance to the predefined inclusion criteria. Twelve full-text articles were retrieved for detailed assessment. All full-texts were successfully obtained and evaluated.

Following full-text review, five studies were excluded as shown in Figure 2. Consequently, seven RCTs [11,12,15,16,17,18,19] involving 554 patients were included in this meta-analysis. Six RCTs [11,16,18,19,20,21] were published as articles and one was available as abstract form only. This RCT was recently reported at the ACC congress 2025 and was recently published as an editorial comment article [17]. All RCTs have some concern regarding the risk of bias as shown in Figure 1 and this was predominantly due to the open-label nature of all these RCTs included. The average age of the patients was 54 years old and only 23% were female (gender not reported by Youssef et al. [15]). 44% had diabetes mellitus, 56% had a history of hypertension and 35% were current smokers. 20% of patients had LVT due to an underlying diagnosis of dilated cardiomyopathy. The follow-up period was 3 months in 5 RCTs [11,13,16,17,18] and 6 months in 2 RCTs [15,19].

### 3.1. LVT Resolution at 3 Months

**Figure 3:**
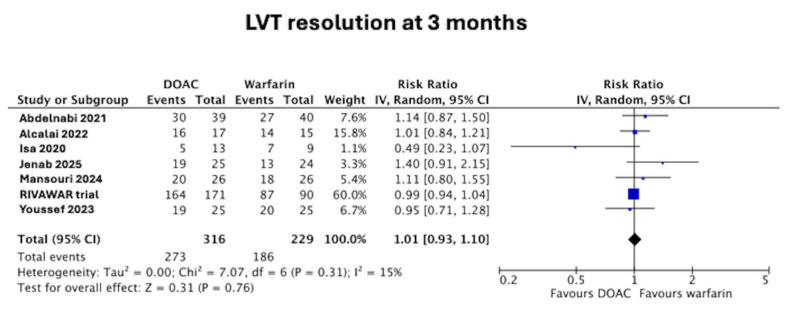
Forest plot of LVT resolution at 3 months in DOAC vs. warfarin treatment groups [11,12,15,16,17,18,19].

LVT resolution data were not available in the trial paper by Isa et al. [19] but these data were subsequently acquired (LVT resolution at 3 months) by contacting the corresponding author [10]. At 3 months, there was no difference in the rate of LVT resolution between those in the DOAC arm and the warfarin arm (86% vs. 81%, RR 1.01 [95% confidence interval (CI) 0.93–1.10], *p* = 0.76), as seen in Figure 3. There was low heterogeneity at I^2^ = 15%.

### 3.2. Major or Clinically Significant Bleeding

**Figure 4:**
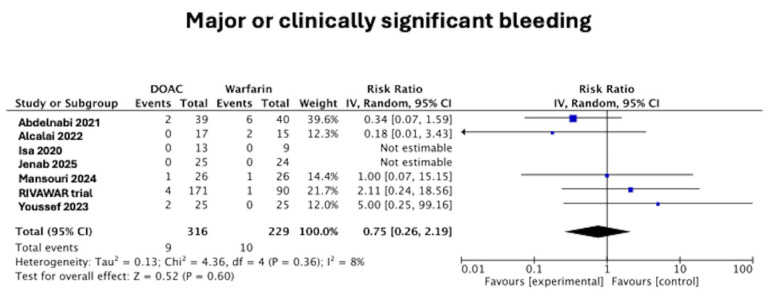
Forest plot of major or clinically significant bleeding in DOAC vs. warfarin treatment groups [11,12,15,16,17,18,19].

Only Youssef et al. [15] explicitly used the Bleeding Academic Research Consortium (BARC) definition. The other trials either did not report a standardized bleeding definition or did not provide detailed safety endpoint methodology. There was no difference in the major or clinically significant bleeding between those in the DOAC arm and the warfarin arm (2.8% vs. 4.4%, RR 0.75 [95%CI 0.26–2.19], *p* = 0.60), as seen in Figure 4. There was low heterogeneity at I^2^ = 8%.

### 3.3. Stroke or Thromboembolic Complications

There was no difference in the composite of stroke or thromboembolic complications between those in the DOAC arm and the warfarin arm (2.2% vs. 3.4%, RR 0.75 [95%CI 0.12–4.25], *p* = 0.72). Heterogeneity was moderate at I^2^ = 40%.

### 3.4. Apixaban Versus Rivaroxaban for LVT Resolution at 3 Months

Three RCTs each compared apixaban against warfarin [15,17,18] and four RCTs compared rivaroxaban against warfarin [11,13,16,19]. Indirect comparison of apixaban versus rivaroxaban showed numerically lower LVT resolution rate with apixaban (40/55, 73%) when compared to rivaroxaban (233/261, 89%) but this was not statistically significant (RR 0.90, 95%CI 0.70–1.15).

## 4. Discussion

In this meta-analysis, we included seven RCTs involving 554 patients with LVT. We found no significant difference in LVT resolution at 3 months between patients treated with DOACs and those treated with warfarin. These findings are aligned with emerging real-world data [19] and add to the growing body of evidence suggesting that DOACs may be a safe and effective alternative to vitamin K antagonists for LVT.

The comparable efficacy observed in LVT resolution supports the biological plausibility that factor Xa inhibition, central to both rivaroxaban and apixaban, is sufficient to prevent thrombus propagation and facilitate resolution [21]. Notably, thrombus resolution was numerically higher in the rivaroxaban-treated patients compared to those treated with apixaban in indirect comparisons, though this difference did not reach statistical significance. This discrepancy could reflect pharmacokinetic differences, patient selection, or trial-level heterogeneity, but should be interpreted cautiously due to the absence of direct head-to-head trials.

Safety outcomes were also similar between groups. The pooled rate of major or clinically relevant bleeding was low and not significantly different between DOACs and warfarin, with low heterogeneity. Only one trial employed a standardized definition for bleeding outcomes, highlighting the need for consistent safety endpoint reporting in future trials. Furthermore, the CIs for both bleeding and embolic outcomes were quite wide, indicating that these RCTs, even after pooling in a meta-analysis, were not adequately powered. Larger RCTs powered for these safety outcomes are warranted.

It is worth noting that a substantial proportion of included patients had LVT secondary to dilated cardiomyopathy, a group for whom anticoagulation strategy remains even less well-defined. None of the RCTs reported disaggregated outcomes for this group. This lack of etiologic subgroup reporting represents a key limitation of the evidence base and of our meta-analysis. The consistency of treatment effects across this heterogeneous population suggests that DOACs may be broadly applicable; however, it is possible that LVT in the setting of DCM may carry different risks of thrombus persistence or embolization. Subgroup-specific data, dedicated trials or patient-level pooled analyses are warranted to clarify optimal anticoagulation strategy in this population.

Notably, recent analyses, including the meta-analysis included in the RCT by Jenab et al. [11], have highlighted the substantial heterogeneity in imaging modalities, timing of follow-up, and reporting of thrombus characteristics across RCTs, factors which may have influenced observed resolution rates. Standardized imaging protocols, consistent definitions of thrombus morphology, and extended follow-up are needed in future trials to clarify these important clinical outcomes. The ongoing RCTs such as the EARLYmyo-LVT trial (N = 280) (NCT03764241), WaRMIN trial (N = 196) (NCT05794399) are using CMR for the assessment of LVT resolution. Rivaroxaban in Left Ventricular Thrombus trial (N = 320) (NCT04970576) is using transthoracic echocardiography (TTE) and the ACTonLVT trial (N = 320) (NCT05892042) is using either TTE or CMR for the assessment of LVT resolution.

### Strengths and Limitations

This is the most comprehensive synthesis to date of randomized data comparing DOACs and warfarin for LVT. A key strength of this analysis is the exclusive inclusion of RCTs, which reduces confounding and selection bias inherent in prior meta-analyses relying heavily on observational data. We also addressed data gaps by contacting authors to obtain unpublished outcomes, improving the completeness and robustness of our pooled analysis.

Nonetheless, several limitations should be acknowledged. First, the total number of patients remains modest, and event rates were low, particularly for safety endpoints, which limits the power to detect small differences. Second, there was heterogeneity in DOAC dosing, background antiplatelet therapy, and imaging modalities used for LVT assessment across trials. Third, the indirect comparison between apixaban and rivaroxaban should be considered hypothesis-generating only and should not guide clinical decision-making in the absence of head-to-head evidence. Fourth, the reliance on one abstract-only trial, open-label design of all the RCTs included and may have introduced bias. Fifth, only one RCT used a standardized definition for bleeding and future trials need to take this into account during the trial design stage, to minimize heterogeneity when pooling future RCTs in this field. Sixth, we performed a study-level rather than a patient-level meta-analysis. Lastly, none of the ongoing RCTs are powered for hard clinical outcomes. Therefore, there is a pressing need for the community to conduct RCTs adequately powered for hard clinical outcomes in this field to provide robust efficacy and safety data.

## 5. Conclusions

In conclusion, this meta-analysis of RCTs demonstrates that DOACs are comparable to warfarin in achieving LVT resolution at 3 months. The meta-analysis was underpowered for safety outcomes but there was no signal of harm. These findings support the consideration of DOACs as a reasonable alternative to VKAs in selected patients with LVT, particularly in settings where warfarin use is logistically challenging. Future adequately powered trials for hard clinical outcomes are needed to inform clinical practice and guideline recommendations.

## Data Availability

The raw data supporting the conclusions of this article will be made available by the authors on request.
